# Genetic Profile in Genes Associated with Cardiorespiratory Fitness in Elite Spanish Male Endurance Athletes

**DOI:** 10.3390/genes12081230

**Published:** 2021-08-10

**Authors:** David Varillas-Delgado, Juan José Tellería Orriols, Juan Del Coso

**Affiliations:** 1 Faculty of Health Sciences, Universidad Francisco de Vitoria, Pozuelo de Alarcon, 28223 Madrid, Spain; 2 Faculty of Medicine, University of Valladolid, 47003 Valladolid, Spain; telleria@med.uva.es; 3 Centre for Sport Studies, Rey Juan Carlos University, 28008 Fuenlabrada, Spain; juan.delcoso@urjc.es

**Keywords:** endurance, physical performance, sports performance, genotype score

## Abstract

Background: most of the research concerning the influence of genetics on endurance performance has been carried out by investigating target genes separately. However, endurance performance is a complex trait that can stem from the interaction of several genes. The objective of this study was to compare the frequencies of polymorphisms in target genes involving cardiorespiratory functioning in elite endurance athletes vs. non-athlete controls. Methods: genotypic frequencies were determined in 123 elite endurance athletes and in 122 non-athletes. Genotyping of *ACE* (rs4340), *NOS3* (rs2070744 and rs1799983), *ADRA2a* (rs1800544 and rs553668), *ADRB2* (rs1042713 and rs1042714), and BDKRB2 (rs5810761) was performed by polymerase chain reaction. The total genotype score (TGS: from 0 to 100 arbitrary units; a.u.) was calculated from the genotype score in each polymorphism. Results: the mean TGS in non-athletes (47.72 ± 11.29 a.u.) was similar to elite endurance athletes (46.54 ± 11.32 a.u., *p* = 0.415). The distribution of TGS frequencies were also similar in non-athletes and elite endurance athletes (*p* = 0.333). There was no TGS cut-off point to discriminate being elite endurance athletes. Conclusions: the genetic profile in the selected genes was similar in elite endurance athletes and in controls, suggesting that the combination of these genes does not determine endurance performance.

## 1. Introduction

Exercise performance is a complex trait resulting in different environmental factors, such as training, nutrition, social status, and gender. However, inherited features, such as genetics, also play a key role in the probability of becoming an elite athlete [1]. This is because genetics might impact muscle and cardiorespiratory function and adaptation to training stimuli, ultimately modifying exercise performance [2]. Recently, it has been shown that at least 120 polymorphisms in target genes are linked to the capacity of being an elite athlete by measuring genotypic frequencies in elite athletes and sedentary population. These studies have found genetic variants that are more prevalent in elite athletes in athletics [3], soccer [4], triathlon [5], and other power-based sports disciplines [1]. However, only a few of these target genes have been directly associated with endurance performance [6,7]. Most of the research on the influence of genetics on endurance performance has been carried out by investigating target genes separately. Nevertheless, the combined influence of several genetic variants, each with a significant contribution, as well as the complex interaction of genetic variants, is likely the best approach to explain individual variations in endurance performance [8]. 

Several polymorphisms reportedly correlating to athlete performance have gained attention; however, inconsistent research design and varying sports make it difficult to ascertain the relevance of these genes to the wider sporting population [9]. Previous investigations have pointed out that the addition of the influence of several polymorphisms by using a total genotype score (TGS), might predict the likelihood of becoming an endurance or power sports elite athlete [10,11]. Thus, the use of investigations that include several genes might increase the probability of explaining the influence of genetics on the different traits associated with exercise performance [12].

Among the candidate genes associated with endurance performance, an insertion (I)/deletion (D) polymorphism in the gene that codifies the angiotensin I-converting enzyme (*ACE*) (rs4340) has been widely studied. Specifically, the insertion (I) rather than deletion (D) is associated with lower circulating angiotensin I-converting enzyme activity and with higher endurance performance [13], although is not always the case [14]. Two polymorphisms have been associated with important phenotypes for endurance performance in the gene that codifies nitric oxide synthase 3 (*NOS3*). The T allele in the *NOS3* c.-786T/C polymorphism (rs2070744) has been related to both power and endurance exercise performance [15] due to enhanced efficiency in the functioning of the athletes’ cardiorespiratory systems during exercise [16]. However, the T allele in c.894G/T polymorphism (rs1799983) is a genetic factor for hypertension [17]. Genotypic variations in the gene that codifies α2a-adrenoceptor (*ADRA2A*) c.-1291C/G (rs553668) and c.1780A/G (rs553668) have been associated with elite endurance athlete status [18], but the information about this gene is scarce and contradictory despite the key role of α2a-adrenoceptor in regulating neurotransmitter release from sympathetic nerves and regulating vascular adaptations to endurance training [19]. In the gene that codifies β-2-adrenergic receptors (*ADRB2*), two polymorphisms (46A/G and 79C/G) promote positive aerobic phenotypes upregulating lipolysis during exercise. However, there is no demonstrable evidence of the predictive ability of this genotype for identifying potential elite athletes [20]. Lastly, the polymorphism −9/+9 in the gene of the bradykinin receptor B2 (*BDKRB2*) has been associated with endurance performance. The absence (−9) of a 9 bp repeat sequence in exon 1 of the *BDKRB2* has been associated with the efficiency of muscular contraction during running [21]. In addition, the −9/−9 genotype was prevalent in elite triathletes compared with a control group [20].

The aim of our research was to compare the frequencies of the polymorphic variations of these target genes involving cardiorespiratory functioning in elite endurance athletes vs. non-athlete controls. By adding the influence of each gene through a total genotype score, we will try to predict the likelihood of becoming an endurance elite athlete by providing a genotype score cut-off point.

## 2. Materials and Methods

### 2.1. Design

A prospective transversal study with case (elite endurance athletes) and controls.

### 2.2. Subjects

We studied 123 elite endurance athletes and 122 non-athlete’s subjects, all of them males. Non-athlete subjects and elite endurance athletes’ three previous generations were of Caucasian descent. Elite endurance runners had a certified high level according to their times (<2 h 10 m in marathon or <1 h 3 min in ½ marathon, or <29 min in 10 k or <14 min in 5 k for runners) or because they had competed in one-day endurance competition or/and Grand Tours (Tour de France, Giro d’Italia and Vuelta a España for cyclists).

All subjects involved in the study signed the informed consent. The study protocol was approved by the Committee of Institutional Ethics of University of Valladolid (protocol code UVa-21/2019) and complied with the Declaration of Helsinki for Human Research of 1974 (last modified in 2003). Participants’ rights and confidentiality were protected during the whole experiment, and the genetic information was used only for the purposes included in this investigation.

### 2.3. Genetic Analyses

We analysed the genetic variants at the Institute of Biology and Molecular Genetics (IBMG), University of Valladolid, Spain. Genomic DNA was obtained from ethylenediaminetetraacetic acid (EDTA) anticoagulated blood samples according to standard phenol-chloroform procedures, followed by precipitation with ethanol. All DNA samples were then stored in the same conditions at −20 °C until subsequent processes were performed. The samples were genotyped using Mastercycler ep gradient S Eppendorf^®^ Thermocycler (Eppendorf, Hamburg, Germany).

#### 2.3.1. ACE Genotyping

The I/D variant (rs4340) of the *ACE* gene was studied by direct genotyping, using forward 5′-CTGGAGACCACTCCCATCCTTTCT-3′ and reverse 5′-GATGTGGCCATCACATTCGGTCAGA-3′ primers. The PCR mixture was denatured at 94 °C for 10 min, amplifying in 35 cycles of 30 s at 94 °C, 30 s at 58 °C, and 1 min at 72 °C, final extension of 7 min at 72 °C. When homozygous individuals were observed for the D (D/D) allele, we performed a second round of amplification to avoid mistyping produced by the D allele that prevents the existence of non-amplified allele I. In this second round was used forward 5′-TGGGACAGCGCCCGCCACTAC-3′ and reverse 5′-TCGCCAGCCCTCCCATGCCCATAA-3′ primers. The PCR mixture was denatured at 94 °C for 10 min, amplifying in 35 cycles of 30 s at 94 °C, 30 s at 67 °C, and 1 min at 72 °C, final extension of 10 min at 72 °C. PCR products were separated through a 2% agarose gel. 

#### 2.3.2. NOS3 Genotyping

The *NOS3* c.-786T/C polymorphism (rs2070744) was genotyped using forward 5′-GAGGTCTCGAAATCACGAGG-3′ and reverse 5′-ATACAAGAACTCCTGGATCC-3′ primers. The PCR mixture was denatured at 95 °C for 5 min, amplifying in 40 cycles of 30 s at 95 °C, 30 s at 60 °C, and 45 s at 72 °C, final extension of 7 min at 72 °C, follow by a restriction using MspI enzyme (Thermo Fisher Scientific, Waltham, MA, USA), separated through a 2% agarose gel. For the *NOS3* c.894G/T polymorphism (rs1799983), a forward 5′-AAGGCAGGAGACAAGTGGATG-3′ and reverse 5′-CAGTCAATCCCTTTGGTGCT-3′ primers were used. The PCR mixture was denatured at 95 °C for 5 min, amplifying in 30 cycles of 1 min at 94 °C, 1 min at 56 °C, and 1:30 min at 72 °C, final extension of 5 min at 72 °C, followed by a restriction by MboI enzyme (Thermo Fisher Scientific, USA), separated through a 2% agarose gel.

#### 2.3.3. ADRA2A Genotyping

The *ADRA2A* c.-1291C/G polymorphism (rs553668) was genotyped with forward 5′-TCACACCGGAGGTTACTTCCCTCG-3′ and reverse 5′-TCCGACGACAGCGCGAGT-3′ primers. The PCR mixture was denatured at 94 °C for 3 min, amplifying in 40 cycles of 30 s at 95 °C, 45 s at 60 °C, and 45 s at 72 °C, final extension of 10 min at 72 °C. After restriction by DraI enzyme (Thermo Fisher Scientific, Waltham, MA, USA), restriction products were separated through a 2% agarose gel. For the *ADRA2A* 1780A/G (rs553668) polymorphism the forward 5′-CAGAGCAGCACTGGACTAC-3′ and reverse 5′-TGGAAGGCATCTCTCCCAAG-3′ primers were used. The PCR mixture was denatured at 95 °C for 5 min, amplifying in 40 cycles of 40 s at 95 °C, 40 s at 60 °C, and 40 s at 72 °C, final extension of 7 min at 72 °C, followed by restriction by DraI enzyme (Thermo Fisher Scientific, Waltham, MA, USA), separated through a 2% agarose gel.

#### 2.3.4. ADRB2 Genotyping

In the *ADRB2* gene, we studied two polymorphisms; c.46A/G Arg16Gly (*ADRB2_R16G_*; rs1042713) and c.79C/G Gln27Glu (*ADRB2_Q27E_*; rs1042714). For both polymorphisms, the forward 5′-GCCTTCTTGCTGGCACCCCAT-3′ and reverse 5′-CAGACGCTCGAACTTGGCCATG-3′ primers were used. The PCR mixture was first denatured at 94 °C for 2 min, amplifying in 40 cycles of 40 s at 94 °C, 40 s at 64 °C, and 50 s at 72 °C, final extension of 7 min at 72 °C. Was used restriction by NcoI (Thermo Fisher Scientific, Waltham, MA, USA) for ADRB2_R16G_ and by BseXI (BbvI) enzymes for *ADRB2_Q27E_* (Thermo Fisher Scientific, Waltham, MA, USA), separated through a 2% agarose gel.

#### 2.3.5. BDKRB2 Genotyping

The I/D +9 pb/−9 pb variant (rs5810761) variant of the *BDKRB2* was genotyped with forward 5′-GCCCTTGAAAGATGAGCTG-3′ and reverse 5′-AACTCCCCACGACCACAG-3′ primers. The PCR mixture and thermal-time profile were first denatured at 94 °C for 5 min, amplifying in 40 cycles of 1 min at 94 °C, 1 min at 53 °C, and 1 min at 72 °C, final extension of 5 min at 72 °C, separated through a 2% agarose gel. 

### 2.4. TGS Determination

The probability that an individual would possess the “optimal” genotype for each of the eight polymorphisms was calculated. We made a scale with the estimated probability of having a “perfect” genetic profile, considering the number of polymorphisms included in this profile [11]. We analysed the combined influence of the eight polymorphisms studied, following the procedure of Williams and Folland [22]. A genotype score (GS) of two was assigned to the “optimal” or preferable endurance genotype, while a GS of 0 was assigned to the less optimal genotype [11] (Table 1). The GSs of all genotypes were added and the score was transformed to 0–100 arbitrary units (a.u.), namely TGS), as follows:

TGS = (GS_ACE_ + GS_NOS3-786_ + GS_NOS3E298D_ + GS_ADRA2A-1291_ + GS_ADRA2A1780_ + GS_ADRB2R16G_ + GS_ADRB2Q27E_ + GS_BDKRB2_) × (100/16) (1)

A TGS of 100 represents a “perfect” profile and a TGS of 0 would be the “worst” profile for endurance sports [22]. The TGSs’ distribution between elite endurance athletes and non-athletes was assessed.

### 2.5. Statistical Analyses

The statistical analysis was carried out using Statistical Package for the Social Sciences (SPSS), v.21.0 for Windows (IBM Corp. Released 2012. IBM SPSS Statistics for Windows, Version 21.0. Armonk, NY: IBM Corp). 

The Hardy–Weinberg equilibrium (HWE) was tested for each polymorphism using χ2 tests. The probability of having an “optimal” endurance genotype for one to eight polymorphisms between endurance elite athletes and non-athletes was calculated using the χ2 test with fixed α 0.05. The genotypic frequencies of the polymorphisms in target-selected genes were compared between elite endurance athletes and non-athletes, using a χ2 test with fixed α 0.05. 

The ability of TGS to correctly distinguish potential elite endurance athletes from non-athletes (0 = non-athlete, 1 = elite endurance athlete) was assessed using receiver operating characteristic (ROC) curves, being calculated the area under the ROC curve (AUC) with confidence intervals of 95% (95%CI). 

A binary logistic regression model was used to study the relationship between TGS and the athletic status.

## 3. Results

**Figure 1:**
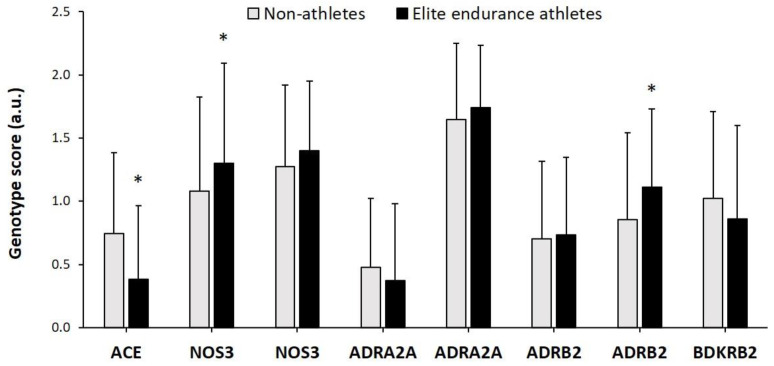
Individual genotype scores in elite endurance athletes and controls, * *p* Value < 0.01.

The individual genotype score for each of the SNPs used in this investigation are presented in Figure 1. Elite endurance athletes had a higher genotype score than non-athletes in the NOS3 c.-786T/C polymorphism (*p* = 0.010) and in the ADRB2_Q27E_ (*p* = 0.003). On the contrary, non-athletes had a higher genotype score than elite athletes in ACE (*p* < 0.001). There were no other between-group differences for ADRA2A variants, NOS3_E298D_, ADRB2_R16G_ nor for BDKRB2. However, the “optimal” genotype score for ADRA2A was higher in elite endurance athletes than non-athletes (Table 1; *p* = 0.016). Genotype frequencies for all polymorphisms were in HWE in both groups. 

**Figure 2:**
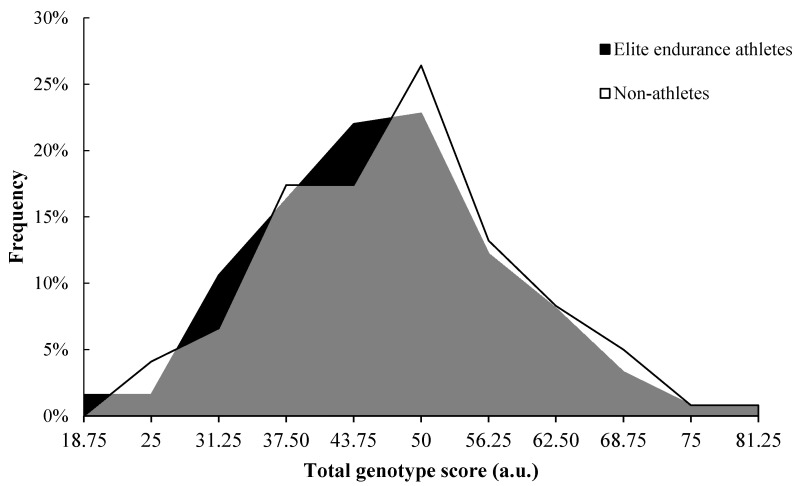
Frequency distribution of total genotype score in elite endurance athletes and controls.

When adding up the individual genotype scores, the mean value of the TGS in non-athletes (47.72 ± 11.29 a.u., statistical kurtosis: −0.01 ± 0.43 a.u.) was similar to elite endurance athletes (46.54 ± 11.32 a.u., statistical kurtosis: 0.24 ± 0.43 a.u.; *p* = 0.415). The distributions of TGS frequencies were also similar in non-athletes and elite endurance athletes (Figure 2; *p* = 0.775). 

Lastly, there were no differences between the accumulative distribution of genotype scores between non-athletes and elite endurance athletes (Table 2; *p* = 0.692).

**Figure 3:**
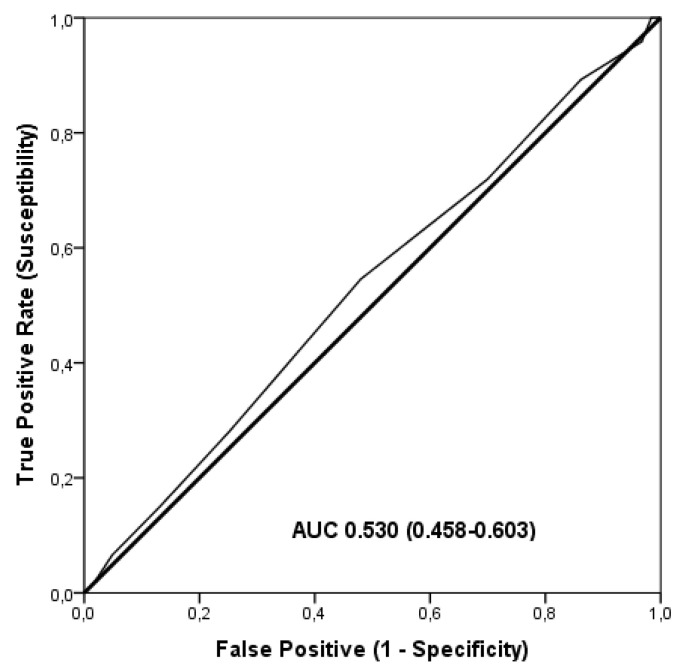
ROC analysis summarizing the ability of TGS to distinguish potential endurance elite athletes from non-athletes.

The discriminatory accuracy of TGSs in the identification of elite endurance athletes was not statistically significant using ROC analysis (AUC = 0.530; 95%CI: 0.458–0.603; *p* = 0.413); sensitivity 0.719, specificity 0.699 (Figure 3). The cut-off point corresponding to TGS value was 40.62 a.u. 

Binary logistic regression analysis showed that subjects with TGS higher to 40.62 a.u. had an odds ratio (OR) of 1.056 (95%CI: 0.611–1.822; *p* = 0.846) of being elite endurance athletes, compared to those with a TGS below (Figure 3). 

## 4. Discussion

To date, several polymorphisms have been associated with endurance performance, but the inconsistency in the research designs and the different phenotypes studied, represented by the diverse nature of endurance sports, make it difficult to ascertain the relevance of these genes to overall performance. In the current study, we sought to increase the power of the analysis of the influence of genetics on endurance performance by simultaneously investigating eight polymorphisms in target genes associated with cardiorespiratory function. Although the genotype score and the frequency distribution of the optimal genotype in *NOS3* 786G/T polymorphism was better in elite endurance athletes than in controls (Figure 1), there were no differences in the TGS between elite endurance athletes vs. non-athlete controls. This information suggests that the addition of the optimal polymorphic variants associated with endurance performance might not offer any benefit for overall endurance performance. Still, it is possible that a greater number of involved genes, codifying for proteins associated with, for example, cardiovascular function, energy metabolism, oxidative stress and recovery of systemic homeostasis, is needed to determine the best possible combination of genetic polymorphisms that allows excelling in endurance sports [23]. In addition, with the current data, it is very unlikely that an individual will be found with an optimal polygenic profile in cardiorespiratory genes to excel in endurance-oriented sports events [24,25].

*ACE* was one of the genes that was initially associated with endurance performance. Montgomery et al. [26] found a very low proportion of elite mountaineers with the *ACE* DD genotype, suggesting that the D allele was somewhat related to reduced endurance performance. Although subsequent investigations found a similar association of the D allele with reduced endurance-like performance [27], more recent studies have disputed any relevance of the *ACE* gene on the likelihood of being an elite endurance athlete [14,28]. The current study confirms this latter scenario, because the proportion of the II genotype or the I allele was lower in elite endurance athletes (Table 1), conferring a lower genotype score compared to non-athlete controls. Thus, the contradictory nature of the research findings in the last 20 years suggest a minor role of *ACE* I/D genotype in elite endurance performance.

The *NOS3* gene encodes a protein involved in coronary artery disease [29,30], while the allele T of c.894G/T polymorphism might induce high blood pressure levels [17]. However, several investigations have found that the T allele of c.-786T/C polymorphism is normally more common in elite athletes or that they have higher values in some variable related to sports performance. In a study with Spanish power athletes, the TT genotype was more prevalent in power-based athletes (57%) than in endurance athletes, while endurance athletes and controls had a similar frequency of TT individuals (~33%) [15]. In our data, endurance athletes had 50.4% of TT individuals, which was higher than non-athlete controls. Although previous studies have associated *NOS3* TT genotype with excel power performance [30,31], the present investigation suggests that this genotype might be linked to the potential of being an elite athlete. This supposition can be made because the frequency of TT in c.-786T/C polymorphism of endurance athletes was comparable to studies with power athletes [15]. Nevertheless, this hypothesis must be confirmed in subsequent studies including athletes of other endurance disciplines such as swimming.

In the current investigation, we sought the genotypic frequency in genes associated with α- and β-adrenergic receptors. Regarding the *ADRA2A* variants, there was no difference in the genotype score, but elite endurance athletes had a higher frequency of the “optimal” genotype in *ADRA2A* c.-1291C/G frequency (5.6% versus 2.5%) compared to non-athlete controls. Only one previous investigation has found a similar association of the *ADRA2A* variants with endurance performance [18] and further investigations should be carried out on this gene due to the role that α-adrenergic receptors have on regulating vascular adaptations to endurance training [19].

In the *ADRB2* gene, we studied two polymorphisms, *ADRB2* Gly16Arg and *ADRB2* and Gln27Glu, as previously investigated in endurance vs. strength/power athletes [32]. In the work by Sawczuk et al., the Gly16 and the Glu27 alleles increased the probability of becoming a strength/power athlete rather than an endurance-oriented athlete. In the current study, there were no between-group differences in the Gly16Arg polymorphism but the frequency of the optimal genotype for the Gln27Glu polymorphism in the sample of elite endurance athletes was higher than in the group of controls, which produced that the individual genotype score for this polymorphism presented a difference in cases vs. controls (Figure 1). Still, the most frequent genotype was CG for both groups indicating that the optimal genotype in the Gln27Glu polymorphism may have a marginal role for the obtaining of the elite endurance athlete status, as previously suggested [20]. 

*BDKRB2* gene encodes bradykinin B2 receptors, which recognise bradykinin; a potent endothelium dependent vasodilator with the capacity of reducing blood pressure [24]. Polymorphism of +9 pb/−9 pb repeat sequence in exon 1 is associated with altered *BDKRB2* messenger ribonucleic acid (mRNA) expression and the +9/+9 genotype is associated with higher systolic and diastolic blood pressures [33]. Specifically, the −9/−9 genotype of the *BDKRB2* gene is overrepresented in endurance athletes compared to male controls [25], results that do not agree with the data in our study, as least in statistically significant terms (Table 1). When comparing 74 Israeli endurance athletes and 240 controls, there was no difference in the frequencies of the −9 allele and −9/−9 genotype between groups [34]. In the same way, the −9/−9 genotype of the *BDKRB2* gene was not more frequent in long distance swimmers [35]. Thus, it seems that +9 pb/−9 pb of the *BDKRB2* gene is poorly associated with being an elite endurance athlete.

This model presents some limitations: (1) the small population sample precludes us from drawing firm conclusions, yet we believe this limitation is justifiable as there are hardly any better endurance specialists in Spain, and it is not easy to reconcile this premise with the scarce number of world-class athletic champions for a given ethnicity and sport event. (2) Numerous genetic variants that have not been included in the model are likely to appear in the foreseeable future, which can also explain individual variations in the potential for attaining elite endurance athletic status. Whereas early genetic studies focused on a single polymorphism, we adopted an exclusive polygenic model. (3) This study has only focused on genetic data, forgetting about genetic markers to associate the genotype found with the phenotype in these athletes, which will need to be completed in future research. (4) Future research is also necessary in women, as the influence of some polymorphisms might differ between sexes.

## 5. Conclusions

The distribution of the TGS, obtained by the addition of eight polymorphisms in genes associated with cardiorespiratory function, was not different in elite endurance athletes when compared to non-athletes in the Caucasian population. This information suggests that the addition of polymorphic variants previously associated with endurance performance might not offer any benefit for the likelihood of becoming an elite endurance athlete, at least in the population studied (Spanish elite endurance runners and cyclists). In fact, the only gene with a positive association with endurance performance was the *NOS3* because more than half of the sample of elite athletes had the optimal genotype. These results suggest the necessity of replicating findings in genetic studies on exercise performance. Lastly, the current investigation raises the need to include epigenetics and environmental aspects in the analysis of the factors associated with elite athlete status. This would improve the understanding of the links between genetic and exercise performance [36]. 

## Figures and Tables

**Table 1:** Studied polymorphisms in elite endurance athletes and non-athletes.

Symbol	Gene	Polymorphism	dbSNP	Genotype Score	Elite Endurance Athletes	Non-Athletes	*p* Value
*ACE*	Angiotensin I-converting enzyme	Alu 287bp (I/D)	rs4340	2 = II	4.88%	10.66%	<0.001
				1 = ID	28.46%	53.28%	
				0 = DD	66.67%	36.07%	
*NOS3*	Nitric Oxide Synthase 3	c.-786T/C	rs2070744	2 = TT	50.41%	31.97%	0.01
				1 = TC	29.27%	44.26%	
				0 = CC	20.33%	23.77%	
*NOS3_E298D_*	Nitric Oxide Synthase 3	c.894G/T	rs1799983	2 = GG	43.09%	38.84%	0.111
				1 = GT	53.65%	51.24%	
				0 = TT	3.25%	9.91%	
*ADRA2A*	α-2a-adrenoceptor	c.-1291C/G	rs1800544	2 = CC	5.61%	2.46%	0.016
				1 = GC	25.23%	42.62%	
				0 = GG	69.16%	54.92%	
*ADRA2A*	α-2a-adrenoceptor	c.1780A/G	rs553668	2 = GG	76.42%	71.07%	0.268
				1 = GA	21.14%	22.31%	
				0 = AA	2.44%	6.61%	
*ADRB2_R16G_*	β-2-adrenergic receptor	c.46A/G	rs1042713	2 = AA	8.94%	8.20%	0.943
				1 = GA	55.28%	54.10%	
				0 = GG	35.77%	37.70%	
*ADRB2_Q27E_*	β-2-adrenergic receptor	c.79C/G	rs1042714	2 = CC	25.20%	17.21%	0.003
				1 = CG	60.98%	50.82%	
				0 = GG	13.82%	31.97%	
*BDKRB2*	Bradykinin Receptor B2	+9 pb/−9 pb	rs5810761	2 = −9/−9	21.14%	24.59%	0.084
				1 = −9/+9	43.90%	53.28%	
				0 = +9/+9	34.96%	22.13%	

**Table 2:** Accumulated percentage through the genotype scores (0–16 a.u.) in elite endurance athletes vs. non-athletes.

Genotype Score	Elite Endurance Athletes	Non-Athletes	*p* Value
0	0 (0.0%)	0 (0.0%)	0.692
1	0 (0.0%)	0 (0.0%)	
2	0 (0.0%)	0 (0.0%)	
3	2 (1.6%)	0 (0.0%)	
4	2 (3.3%)	5 (4.1%)	
5	13 (13.8%)	8 (10.7%)	
6	20 (30.1%)	21 (28.1%)	
7	27 (52.0%)	21 (45.5%)	
8	28 (74.8%)	32 (71.9%)	
9	15 (87.0%)	16 (85.1%)	
10	10 (95.1%)	10 (93.4%)	
11	4 (98.4%)	6 (98.3%)	
12	1 (99.2%)	1 (99.2%)	
13	1 (100.0%)	1 (100.0%)	
14	-	-	
15	-	-	
16	-	-	

## Data Availability

Not applicable.
